# Integrative analysis of metabolites and microbial diversity revealed metabolic mechanism of coarse feeding tolerance in Songliao Black sows during gestation

**DOI:** 10.3389/fmicb.2024.1484134

**Published:** 2024-11-19

**Authors:** Jinbo Zhao, Wenjiang Zhao, Jiaqiang Dong, Hong Zhang, Kun Yang, Shengyue Gao, Wanyu Feng, Yan Song, Meiyu Qi, Xinmiao He

**Affiliations:** ^1^Branch of Animal Husbandry and Veterinary, Heilongjiang Academy of Agricultural Sciences, Qiqihar, China; ^2^Institute of Animal Husbandry, Heilongjiang Academy of Agricultural Sciences, Haerbin, China

**Keywords:** dietary fiber, metabolites, microbial community composition, Songliao sow, coarse feeding tolerance

## Abstract

Dietary fiber is a key nutritional regulatory factor that has been studied intensively for its role in improving reproduction in sows during gestation. However, the metabolic mechanism underlying the effect of interactions between metabolites and gut microbes on coarse feeding tolerance in indigenous sows remains to be elucidated. Therefore, the present study aimed to investigate the effects of dietary supplementation with alfalfa at different content ratios on the reproductive performance of pregnant Songliao Black sows. In total, 40 Songliao Black sows at 30 days of gestation were allocated to four treatments, which received the following diets: (1) a corn–soybean meal basal diet with no alfalfa meal (CON group), (2) a corn–soybean meal basal diet +10% alfalfa meal (Treatment 1 group), (3) a corn–soybean meal basal diet +20% alfalfa meal (Treatment 2 group), and (4) a corn–soybean meal basal diet +30% alfalfa meal (Treatment 3 group). Untargeted metabolomics, 16S rDNA sequencing, and enzyme-linked immunosorbent assay (ELISA) were performed to determine the possible effects of metabolites, the microbial communities in fecal samples and their functional potential, and the effects of dietary fiber on serum biochemical parameters, oxidative stress, and reproductive hormones in Songliao Black sows during gestation. The results revealed that the meals with 10 and 20% alfalfa had a beneficial effect on sows in terms of improving the reproductive performance of these sows. Bacterial 16S rDNA sequencing of the fecal samples revealed that the 10% alfalfa meal group had a higher *α*-diversity and higher abundance of probiotics. Bacteroidetes, Firmicutes, Proteobacteria, and Actinobacteria were revealed as the most abundant groups at the phylum level and *Lactobacillus*, *Prevotella*, *Ruminococcus*, *Streptococcus*, and *Clostridium* were the most abundant at the genus level in the sows fed with diets containing higher fiber levels. A total of 239 differential metabolites were identified in the sows fed with alfalfa meals. These metabolites were enriched mainly in the cAMP signaling pathway, biosynthesis of amino acids, and steroid biosynthesis. Pearson correlation analysis revealed significant positive correlations between Blautia and Daizein, *Fibrobacter* and 5-alpha-Cholestanone, Sphaerochaeta, Sutterella, and Metaraminol. Negative correlations were revealed between *Sphaerochaeta* and Erucic acid, *Prevotellaceae* and Harmaline, and *Streptococcus* and 5-alpha-Cholestanone. Collectively, these findings provide novel insights into the application of dietary fiber in sow diets.

## 1 Introduction

Songliao Black sows are a recently developed synthetic breed of sows in Northern China, which exhibit the beneficial characteristics of coarse feeding tolerance (Ma et al., 2022b), delicious meat quality (Xia et al., 2022), and excellent reproductive performance compared to imported pigs (Li et al., 2016). Consequently, Songliao Black sows are popular throughout China. Mounting evidence suggests that the adaptation and use of coarse fodder in the diets of indigenous pigs depends on, in addition to host genetics, the composition of gut microbiota (Ngoc et al., 2012; Jarrett and Ashworth, 2018; Cuevas-Sierra et al., 2019; Chassé et al., 2022; Ma et al., 2022a). Various studies have indicated that the microbiome in the gastrointestinal tract, which comprises dense populations of trillions of symbiotic bacteria, fungi, archaea, and viruses (Guo et al., 2022), may be induced by a complex variety of factors, such as nutrition (Salazar et al., 2017), environment (Guo et al., 2021), and disease (Zafar and Saier, 2021). Dietary fiber is a vital component of animal nutrition. Gut microbiota decomposes dietary fiber into short-chain fatty acids (SCFAs), which serve as key bacterial metabolites that inhibit the activity of histone deacetylases, resulting in chromatin changes that are generally associated with increased expressions of certain target genes (Koh et al., 2016; Bubier et al., 2021; Woo and Alenghat, 2022).

Alfalfa meal is an unconventional feed resource used commonly in the pig industry, particularly for pregnant sows (Xu et al., 2022). The use of low-opportunity cost feed products (LCFs), such as food waste and by-products, in the feed of animals, is expected to decrease food–feed competition for cropland (Fang et al., 2023). In addition, LCFs would save feed resources, reducing the competition for food between humans and animals. Mechanistically, the metabolites generated upon the decomposition of dietary fiber by gut microbiota could serve as epigenetic regulators, thereby affecting the reproductive performance of animals during gestation. Several studies have, therefore, explored the role of dietary fiber on host physiology, such as the therapeutic intervention for type 2 diabetes (Huda et al., 2021), gastrointestinal health and disease (Gill et al., 2021), and sleep and mental health (Yan et al., 2020). Recently, with rapid advancement in microbiome analysis technology, 16 s rDNA sequencing has emerged as an important research tool, which has been applied widely to study microbial community composition and distribution (Korpela et al., 2020; Zhou et al., 2021). Metabolomics is considered a bridge between genomics and phenotypes, and has, therefore, been applied widely to the fields of disease diagnosis, biomedicine, and toxicology (Fiehn, 2022; Cui et al., 2018; Bujak et al., 2015). However, studies exploring the role of dietary fiber on the reproductive performance of sows through the potential interactions between its catabolic metabolites and gut microbiota are scarce.

Therefore, the present study aimed to investigate the metabolic mechanism underlying the improvement effect of dietary fiber on sow reproductive performance through interactions between its metabolites and gut microbiota using untargeted metabolomics and microbiome analysis. The findings of the study are expected to provide indispensable theoretical support to the research aimed at deciphering the metabolic mechanism of coarse feeding tolerance in Songliao sow during gestation.

## 2 Materials and methods

### 2.1 Ethics statement

The present study was conducted in accordance with the National Institutes of Health guidelines for the care and use of laboratory animals. All study procedures were approved by the Institutional Animal Care and Use Committee of Heilongjiang Academy of Agricultural Sciences (HAAS20140025).

### 2.2 Animals, experimental design, and diets

A total of 40 Songliao Black sows in gestation were selected based on synchronized estrus, similar body weights (initial BW: 230.5 ± 10.5 kg), uniform body condition, and consistent parity (average parity: 3.29). The 40 Songliao Black sows were randomly allocated to four groups: the CON group, which received a corn–soybean meal basal diet without alfalfa meal (*n =* 10); Treatment 1, which received a corn-soybean meal basal diet +10% alfalfa meal (*n* = 10); Treatment 2 which received a corn-soybean meal basal diet +20% alfalfa meal (*n* = 10); and Treatment 3, which received a corn-soybean meal basal diet +30% alfalfa meal (*n* = 10). Dietary treatments began 30 days into gestation and continued until parturition. All sows were housed in individual stalls (250 × 85 × 120 cm). All experimental animals were fed in the same housing environment, with the temperature maintained at 23 ± 2°C and the average relative humidity controlled at 60 ± 5%. All diets were formulated to meet the nutrient requirements of gestation sows documented by the National Academy Press (National Research Council, U.S., 2012). The ingredients and compositions of all diets for sows at gestation are listed in Table 1.

**Table 1:** Composition of the diets fed to pregnant sows in the present study.

Feed compostion	Control group	Treatment 1	Treatment 2	Treatment 3
Corn (%)	64.36	62.83	51.97	53.90
Soybean meal (10%)	17.10	17.44	15.47	10.17
Alfalfa meal (%)	/	10.00	20.00	30.00
Wheat middling	5.00	5.00	5.00	5.00
Wheat bran (%)	9.68	/	/	/
Soybean oil (%)	/	1.72	4.52	20.50
Limestone (%)	1.34	0.92	0.54	0.63
Calcium (%)	1.11	1.13	1.10	1.40
Salt (%)	0.40	0.40	0.40	0.30
Premix (%)	1.00	1.00	1.00	1.00
Total (%)	100	100	100	100
Nutrition level				
DE (MJ/Kg)	12.97	12.97	12.97	12.97
CP (%)	15.00	15.00	15.00	15.00
CF (%)	3.07	4.99	7.12	6.10
Ca (%)	0.81	0.81	0.81	0.81
TP (%)	0.58	0.52	0.49	0.50
AP (%)	0.41	0.41	0.41	0.41
EE (%)	3.06	4.16	7.22	5.69
NaCl (%)	0.55	0.60	0.56	0.62
Lysein (%)	0.70	0.73	0.73	0.73
Methionine (%)	0.24	0.25	0.24	0.24
Tryptophan (%)	0.18	0.20	0.21	0.20
Threonine (%)	0.58	0.61	0.61	0.61

### 2.3 Sample collection and preparation

Fresh feces and blood samples were collected from all four treatment groups at day 110 of gestation. A total of 40 fresh fecal samples were collected from the 40 sows and immediately immersed in liquid nitrogen, then stored at −80°C until used for subsequent untargeted metabolomics analysis. Blood from the anterior vena cava was collected in a tube containing an anticoagulant and then centrifuged at 3000 r/min for 15 min to obtain the serum. All serum samples were stored immediately at −20°C until used for the subsequent serum biochemical parameter analysis.

### 2.4 Reproductive performance parameter analysis

The reproductive performance index was calculated to determine the weight gain of each piglet at birth and the average litter weight at 20 days.

### 2.5 Serum biochemical parameter analysis

The serum levels of follicle-stimulating hormone (FSH), luteinizing hormone (LH), estradiol (E2), and progesterone (P4) were determined for all 40 sows using an ELISA kit according to the manufacturer’s instructions (Jiangsu Mei Mian Industrial Co. Ltd., Taizhou, China). The serum triglyceride cholesterol (TC), low-density lipoprotein cholesterol (LDL-C), glucose (GLU), superoxide dismutase (SOD), and glutathione (GSH) levels were determined through a microplate test. The serum malondialdehyde (MDA) levels were determined using the thiobarbituric acid (TBA) method.

### 2.6 Liquid chromatography–mass spectrometry (LC–MS) to identify the differential metabolites and metabolic pathways

The LC analysis was performed on a Vanquish UHPLC System (ThermoFisher Scientific, USA) using an ACQUITY UPLC^®^ HSS T3 column (150 × 2.1 mm, 1.8 μm) (Waters, Milford, MA, USA). The column was maintained at a temperature of 40°C. The flow rate and injection volume were set to 0.25 mL/min and 2 μL, respectively. In the LC-ESI (+)-MS analysis, the mobile phase comprised 0.1% (v/v) formic acid in acetonitrile (B2) and 0.1% (v/v) formic acid in water (A2). Separation was performed using the following gradient: 0–1 min, 2% B2; 1–9 min, 2–50% B2; 9–12 min, 50–98% B2; 12–13.5 min, 98% B2; 13.5–14 min, 98%–2% B2; 14–20 min, 2% B2. The LC-ESI (−)-MS analysis was conducted using acetonitrile (B3) and 5 mM ammonium formate (A3). Separation was performed using the following gradient: 0–1 min, 2% B3; 1–9 min, 2–50% B3; 9–12 min, 50–98% B3; 12–13.5 min, 98% B3; 13.5–14 min, 98%–2% B3; 14–17 min, 2% B3 (Ma et al., 2022b). The mass spectrometry-based detection of metabolites was performed using Orbitrap Exploris 120 (Thermo Fisher Scientific, USA) equipped with an ESI ion source. Simultaneous MS1 and MS/MS (Full MS-ddMS2 mode, data-dependent MS/MS) acquisition was performed using the following parameters: sheath gas pressure at 30 arb; auxiliary gas flow at 10 arb; spray voltage of 3.50 kV and −2.50 kV for ESI(+) and ESI(−); capillary temperature set to 325°C; an MS1 range of m/z 100–1,000 with appropriate resolving power.

### 2.7 Bacterial 16S rDNA sequencing for fecal samples

Total genomic DNA was extracted from the fecal samples using the cetyltriethylammonium bromide (CTAB) method. The extracted DNA was quantified using Nanodrop. The quality of DNA extraction was evaluated using 1.2% agarose gel electrophoresis. The 16S rDNA genes of distinct regions (16S V3–V4) were amplified using specific primers. Sequencing libraries were generated using TruSeq Nano DNA LT Library Prep Kit. Microbial communities were identified using MiSeq Reagent Kit V3.

### 2.8 Statistical analysis

The result data were expressed as mean ± standard deviations (SD) and analyzed statistically using SPSS 19.0 (IBM Corporation, Armonk, NY, USA). Bars with * and ** represent significance levels of *p* < 0.05 and *p* < 0.01, respectively. The raw data were first converted to the mzXML format using MSConvert in the ProteoWizard software package (v3.0.8789) (Rasmussen et al., 2022) and then processed using XCMS (Navarro-Reig et al., 2015) for feature detection, retention time correction, and alignment. Metabolites were identified by comparing accurate mass measurements (<30 ppm) and MS/MS data with databases including HMDB (Wishart et al., 2007) (http://www.hmdb.ca, accessed on 9 September 2022, MassBank) (Horai et al., 2010) (http://www.massbank.jp/, accessed on 10 September 2022, LipidMaps) (Sud et al., 2007) (http://www.lipidmaps.org, accessed on 10 October 2022), mzCloud (Abdelrazig et al., 2020) (https://www.mzcloud.org, accessed on 10 October 2022, and KEGG) (Ogata et al., 1999) (http://www.genome.jp/kegg/, accessed on 10 November 2022). Data normalization was performed by applying robust LOESS signal correction (QC-RLSC) (Gagnebin et al., 2017) to eliminate any systematic bias. After normalization, only the ion peaks with relative standard deviations (RSDs) <30% in QC were retained to ensure proper metabolite identification. The R language ropes package (Ropls) (Thévenot et al., 2015) software was employed for all multivariate data analyses and modeling. After data scaling, models were constructed based on the principal component analysis (PCA). Finally, the metabolites with a *p* < 0.05 and a VIP value of >1 were considered statistically significant.

The identified differential metabolites were subjected to pathway analysis using MetaboAnalyst (Xia and Wishart, 2011) (v 4.0), which combines the results of a powerful pathway enrichment analysis and those of pathway topology analysis. The metabolites identified in the metabolomics analysis were then mapped to the KEGG pathway for the biological interpretation of higher-level systemic functions. Finally, the metabolites and the corresponding pathways were visualized using the KEGG Mapper tool.

## 3 Results

### 3.1 Effect of dietary fiber with different proportions of alfalfa on the reproductive phenotype parameters and reproductive hormone levels in the serum of sows

**Figure 1:**
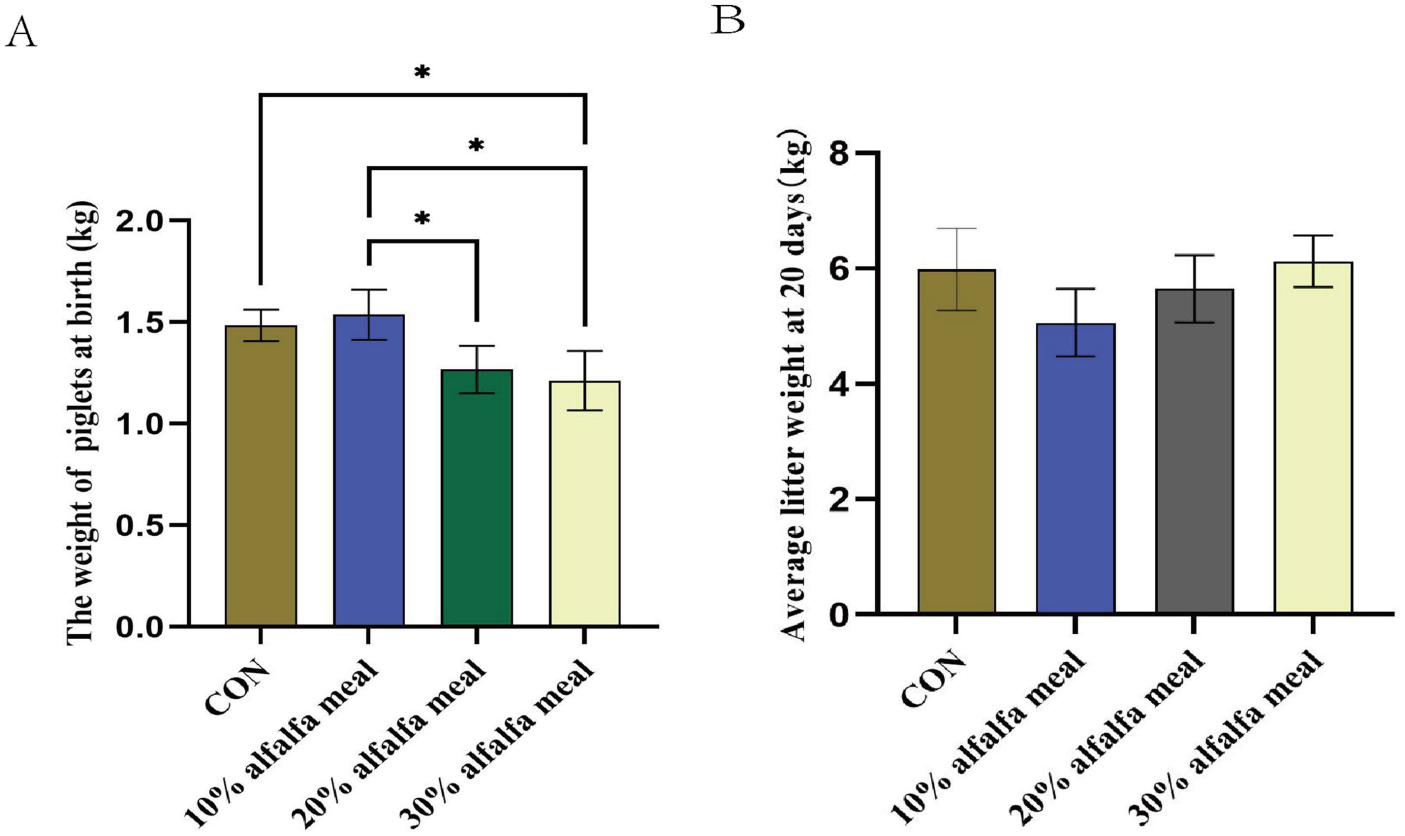
The reproductive performance of sows fed meals with different level of alfalfa. **(A)** The weight of piglets at birth fed with different level of alfalfa. **(B)** Average litter weight at 20 days fed with different level of alfalfa, the diet with 10% alfalfa referred as the Treatment 1 group, the diet with 20% alfalfa referred as the Treatment 2 group, the diet with 30% alfalfa referred as the Treatment 3 group.

Dietary fiber is the indigestible portion of foods derived from plants, and it reportedly affects a wide range of physiological processes, such as behavior and welfare (Sun et al., 2015), reduction in the circulating estradiol (Jarrett and Ashworth, 2018), and a decrease in the circulating metabolites (Weaver et al., 2013). In the present study, the weight gain of piglets at birth and the average litter weight at 20 days were determined during the lactation period. It was revealed that the weight gain of piglets at birth was higher (*p <* 0.05) in the 10% alfalfa meal groups than in the CON group. The average litter weight at 20 days, on the other hand, did not differ significantly compared to the CON group in any of the treatment groups (*p >* 0.05) (Figures 1A,B).

**Figure 2:**
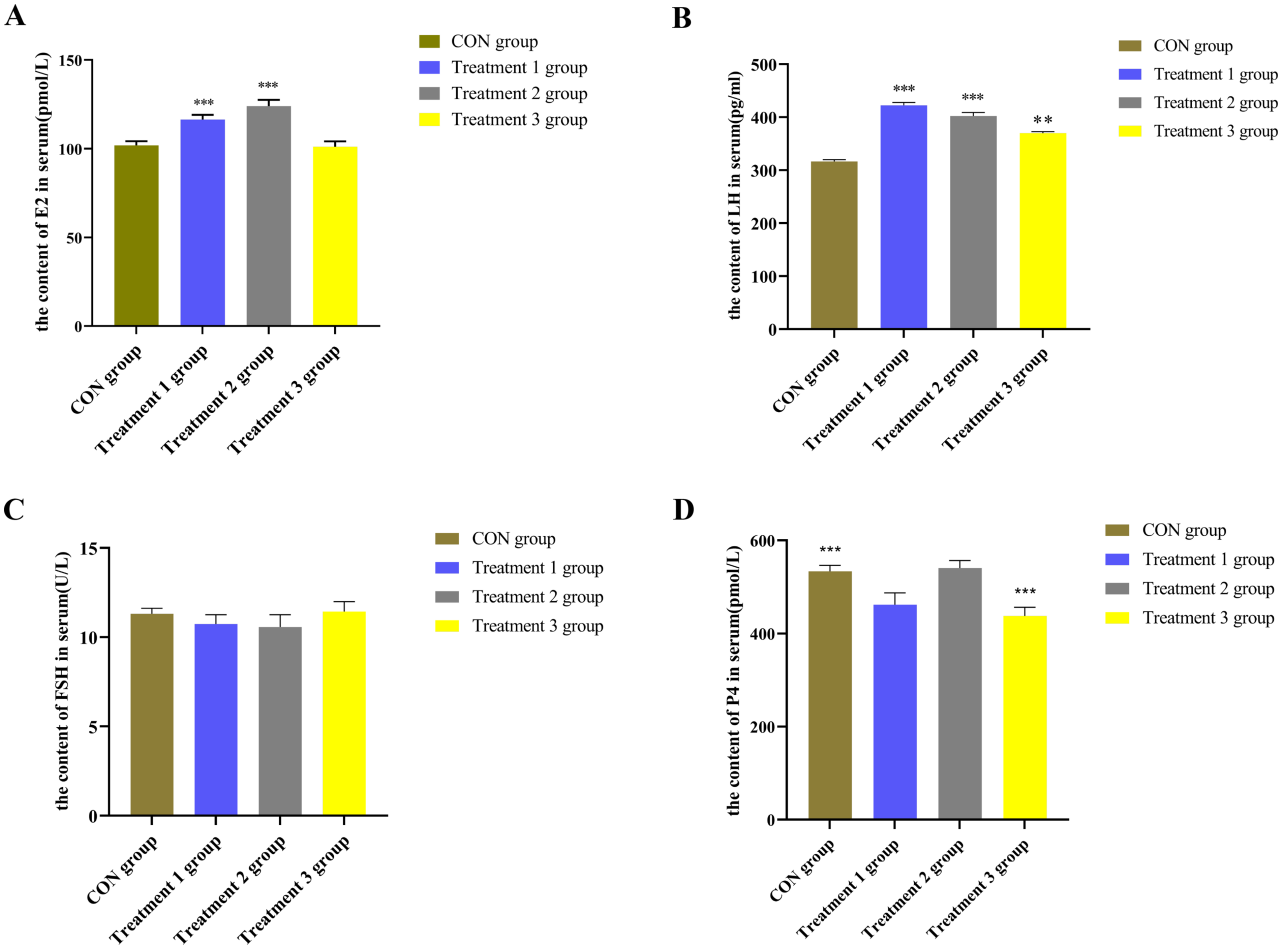
The levels of reproductive hormones in the serum of sows. **(A)** the content of E2 in serum; **(B)** the content of LH in serum; **(C)** the content of FSH in serum; **(D)** the content of P4 in serum. The CON group along the abscissa represents the diet with no alfalfa, the Treatment 1 group represents the diet with 10% alfalfa, the Treatment 2 group represents the diet with 20% alfalfa, and the Treatment 3 group represents the diet with 30% alfalfa. The ordinate presents the levels of different reproductive hormones in the serum of sows. Bar with ** and *** represent significance levels of *p* < 0.01 and *p* < 0.001.

As shown in Figure 2B, the content of LH was significantly higher in the serum samples from the 10 and 20% alfalfa meal groups than in the no alfalfa meal group (*p <* 0.01). The content of P4 was significantly lower in the 20% alfalfa meal group than in the no alfalfa meal group (*p <* 0.01) (Figure 2D). The content of FSH in the 10, 20, and 30% alfalfa meal groups was not significantly different from that in the CON group (Figure 2C). The sows fed with 10% alfalfa meal exhibited increased serum levels of E2 (*p <* 0.01), while the CON group and 30% alfalfa meal group sows presented no significant differences (*p >* 0.05) in the serum levels of E2 (Figure 2A). Collectively, these findings demonstrated that diets with a higher fiber content increase the concentrations of LH and E2 in the serum of sows. Accordingly, it was speculated that dietary fiber could increase the levels of reproductive hormones, which are vital for maintaining sow pregnancy during the late gestation stage.

### 3.2 Effect of dietary fiber with different proportions of alfalfa on oxidative stress and serum biochemical parameters in sows

**Figure 3:**
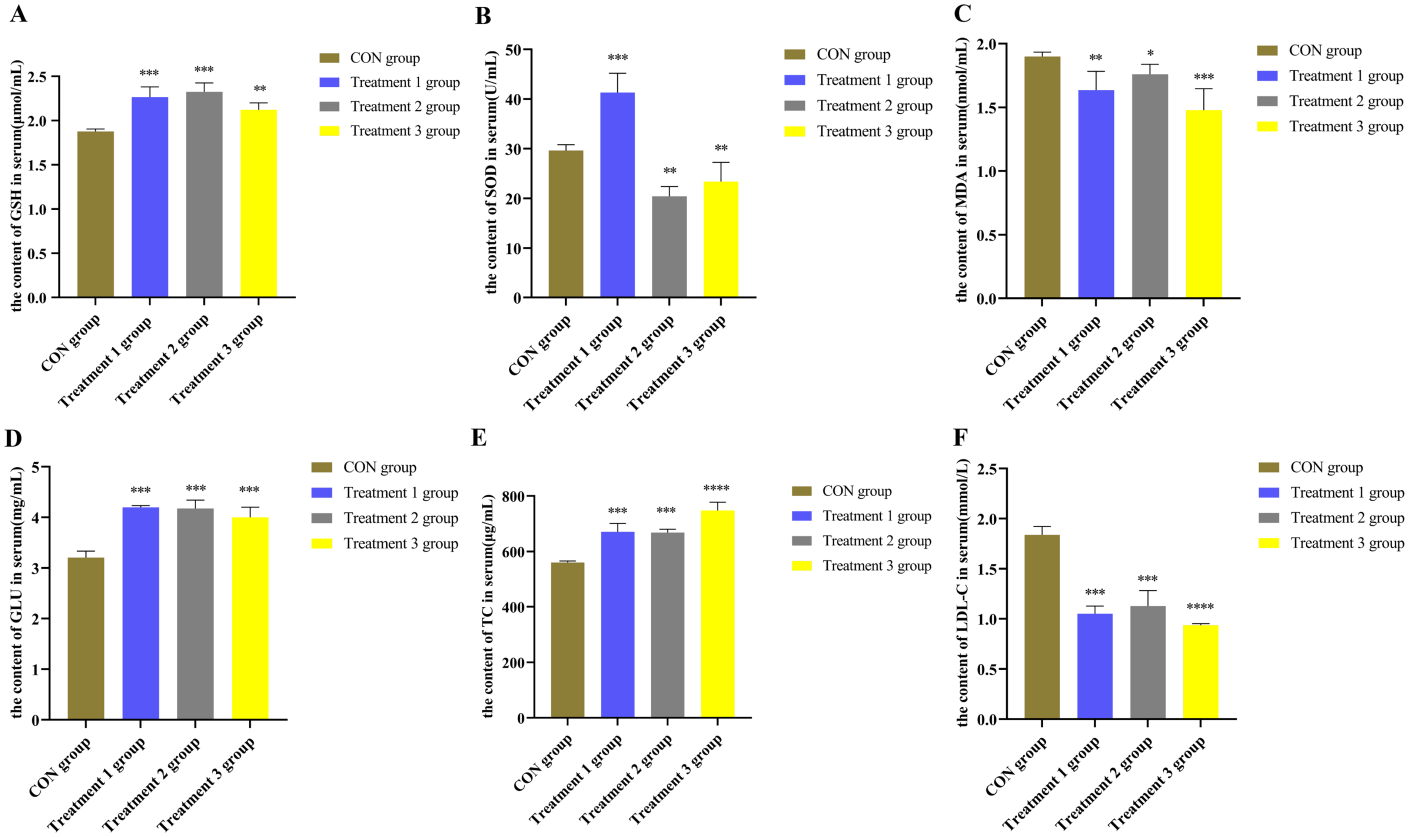
**(A–C)** The levels of the antioxidant indicators in the serum of sows. **(D–F)**. The levels of different biochemical indicators in the serum of Songliao Black sows in the CON group representing the diet with no alfalfa, Treatment 1 group representing the diet with 10% alfalfa, Treatment 2 group representing the diet with 20% alfalfa, and Treatment 3 group representing the diet with 30% alfalfa. Bar with ** and *** represent significance levels of *p* < 0.01 and *p* < 0.001. Bar with **** represent significance levels of *p* < 0.0001.

Sows have an increased demand for energy and oxygen in the placenta during late gestation, which leads to excessive oxidative stress that negatively affects both the mother and the placenta (Yang et al., 2023). Therefore, the effects of various feeds with different proportions of dietary fiber on the serum MDA, SOD, and GSH levels in sows during pregnancy were investigated in the present study. Biochemical tests were performed to determine the antioxidant levels in the serum of these sows. According to the results presented in Figures 3A–C, sows in the 10, 20, and 30% alfalfa meal groups presented significantly increased concentrations of GSH and SOD compared to those in the CON group (*p <* 0.01; *p <* 0.001), while the MDA levels were decreased in the former groups (*p <* 0.05; *p <* 0.01). Interestingly, the sows in the 30% alfalfa meal groups had increased serum levels of GSH compared to the other groups (*p <* 0.01). Accordingly, it was speculated that diets with a higher fiber content might be facilitating the maintenance of the antioxidant system in sows, while diets with 10 and 20% alfalfa could be involved in maintaining the physiological homeostasis of sows.

According to the results presented in Figures 3D–F, sows in the 10 and 20% alfalfa meal groups presented higher concentrations of glucose and TC during late gestation than the sows in the CON group (*p <* 0.001). Moreover, the sows in the 30% alfalfa meal groups presented lower concentrations of LDL-C (*p <* 0.001). The LDL-C levels in the sows fed with 10, 20, and 30% alfalfa meals were significantly decreased (*p <* 0.01) compared to those noted in the CON group sows.

### 3.3 Identification of potential differential metabolites in the fecal samples from Songliao Black sows

**Figure 4:**
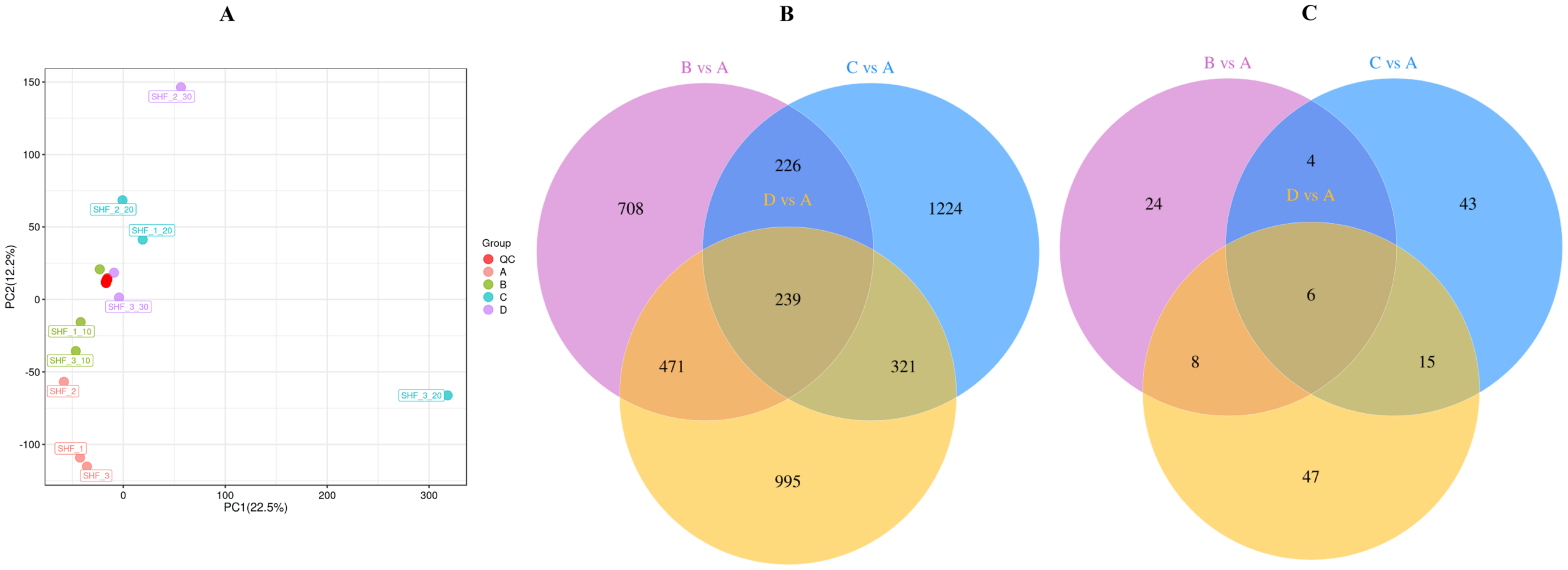
**(A)** The principal component analysis (PCA) plot. **(B,C)** Venn diagram presenting the number of unique and common differential metabolites between different groups. CON group/A, diet with no alfalfa; B, diet with 10% alfalfa; C, diet with 20% alfalfa; D, diet with 30% alfalfa.

The PCA plot depicted in Figure 4A presents a distinct separation between the CON group and the other groups, indicating the accuracy and reliability of the data.

The Venn diagrams presented in Figures 4B,C present the 1,644 common metabolites identified between A (CON group with no alfalfa) and B (10% alfalfa meal group) in the untargeted metabolomics analysis. In addition, 2,010 common metabolites between A and C (20% alfalfa meal group) and 2,026 common metabolites between A and D (30% alfalfa meal group) were identified. A total of 239 metabolites were identified to be shared among A, B, C, and D. Further analysis revealed 42 metabolites common between A and B, 68 metabolites common between A and C, and 76 metabolites shared between A and D. Only 6 metabolites were shared among A, B, C, and D.

**Figure 5:**
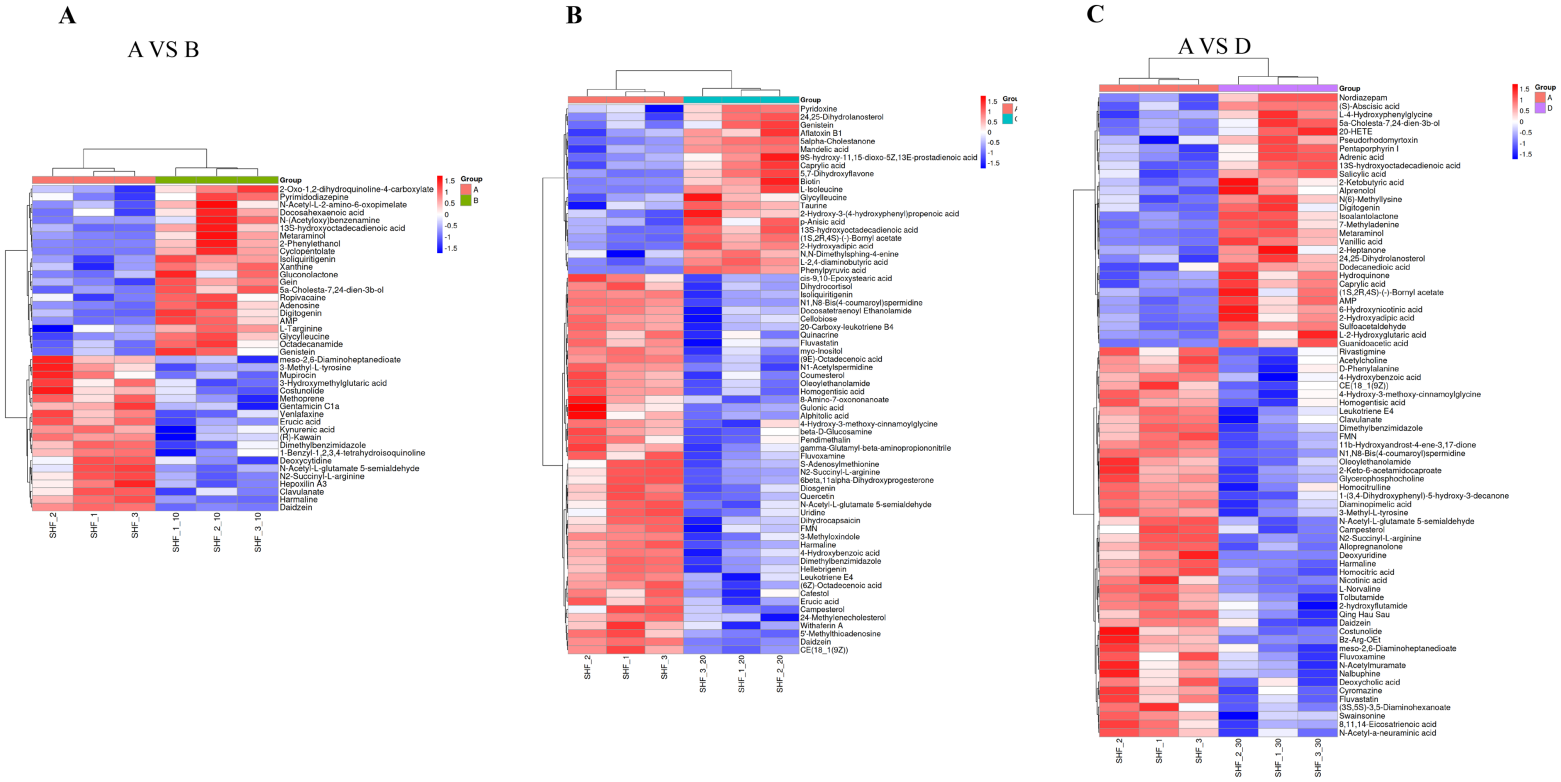
Heatmap presenting the distribution of the identified differential metabolites. **(A)** Presents the results for the 10% alfalfa meal group (B) vs. the CON group (A). **(B)** Presents the results for the 20% alfalfa meal group (C) vs. the CON group (A). **(C)** presents the results for the 30% alfalfa meal group (D) vs. the CON group (A).

Figure 5A illustrates that gein and isoliquirtigenin were upregulated while erucic acid, harmaline, and daidzein were downregulated in the 10% alfalfa meal group compared to the CON group (VIP 1.0). Figure 5B illustrates that L-hydroxyadipic acid, L-2,4-diaminobutyric acid, and 5 alpha-cholestanone were unaltered while Daidzein and N1,N8-bis(4-coumaroy). Spermidine were downregulated in the 20% alfalfa meal group compared to the CON group (VIP 1.0). As visible in Figure 5C, metaraminol and sulfoacetaldehyde were unaltered while L-norvaline, N1,N8-bis(4-coumaroy) spermidine, and harmaline were downregulated in the 30% alfalfa meal group compared to the CON group (VIP 1.0). These results revealed complex metabolic network regulation mechanisms underlying the effects of dietary fiber supplementation on the reproductive performance of sows during late pregnancy.

### 3.4 Differential metabolic pathway analysis of the metabolites identified in the fecal samples from Songliao Black sows

**Figure 6:**
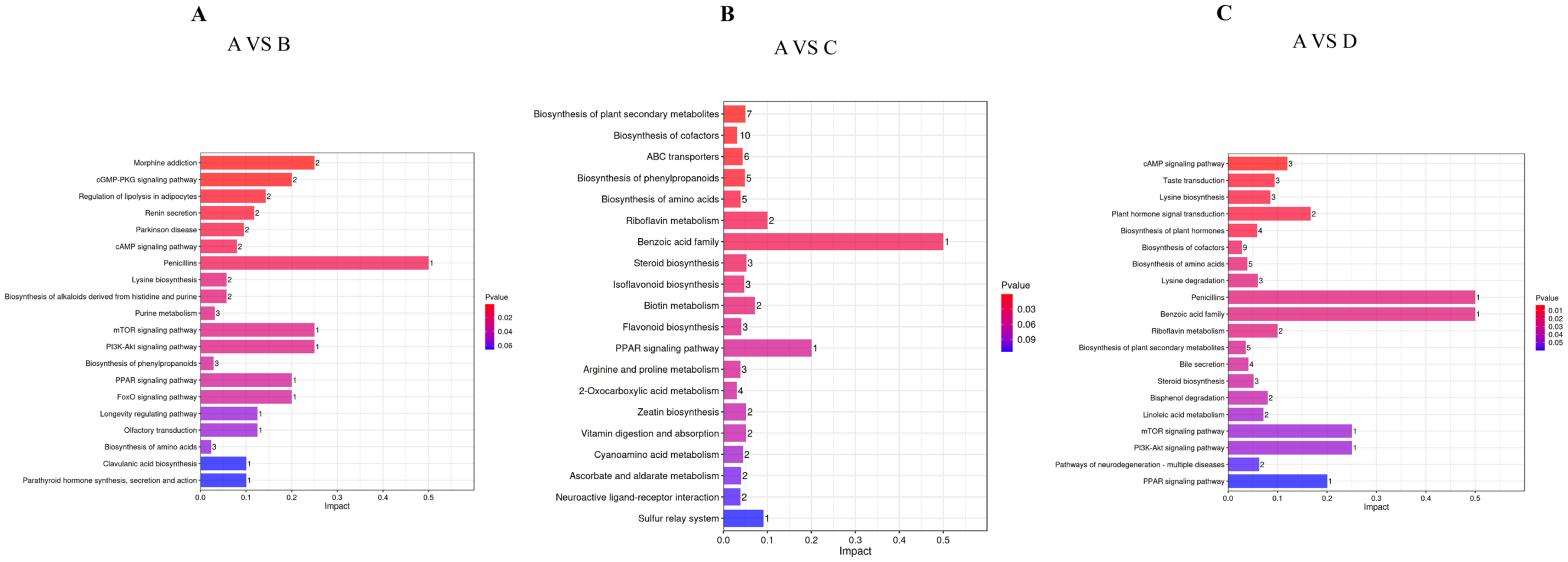
Bar diagram presenting the differential metabolites that were identified through a query in the KEGG database. **(A)** Presents the 10% alfalfa meal group (B) vs. CON group (A). **(B)** Presents the 20% alfalfa meal group (C) vs. CON group (A). **(C)** Presents the 30% alfalfa meal group (D) vs. the CON group (A).

The metabolomic profiles of Songliao Black sows revealed gein and isoliquirtigenin as the upregulated metabolites and erucic acid, harmaline, and daidzein as the downregulated metabolites in the 10% alfalfa meal group (B) compared to the CON group (A). The pathway analysis of these metabolites revealed their enrichment in Morphine addiction-related pathways, the cGMP-PKG signaling pathway, the regulation of lipolysis in adipocytes, renin secretion, Parkinson disease-related pathways, and the cAMP signaling pathway (Figure 6A). The six differential metabolites in the 20% alfalfa meal group compared to the CON group were enriched mainly in the biosynthesis pathways for plant secondary metabolites, cofactors, ABC transporters, phenylpropanoids, and amino acids (Figure 6B). In addition, five metabolites were enriched in the cAMP signaling pathway, taste transduction, lysine biosynthesis, biosynthesis of plant hormones, and plant hormone signal transduction. These results suggested that the dietary fiber intake level mediated energy and fat metabolism to regulate physiological homeostasis, leading to the adaptation of coarse feed tolerance in sows (Figure 6C).

### 3.5 Microbial community composition and diversity in the fecal samples from Songliao Black sows

**Figure 7:**
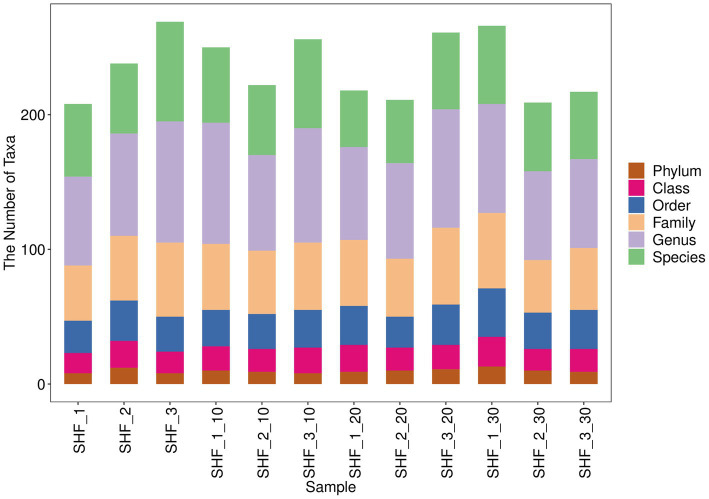
Display diagram of the differential taxonomic units between groups based on the classification rank tree for the 10% alfalfa meal group (B), CON group (A), 20% alfalfa meal group (C), and 30% alfalfa meal group (D).

**Figure 8:**
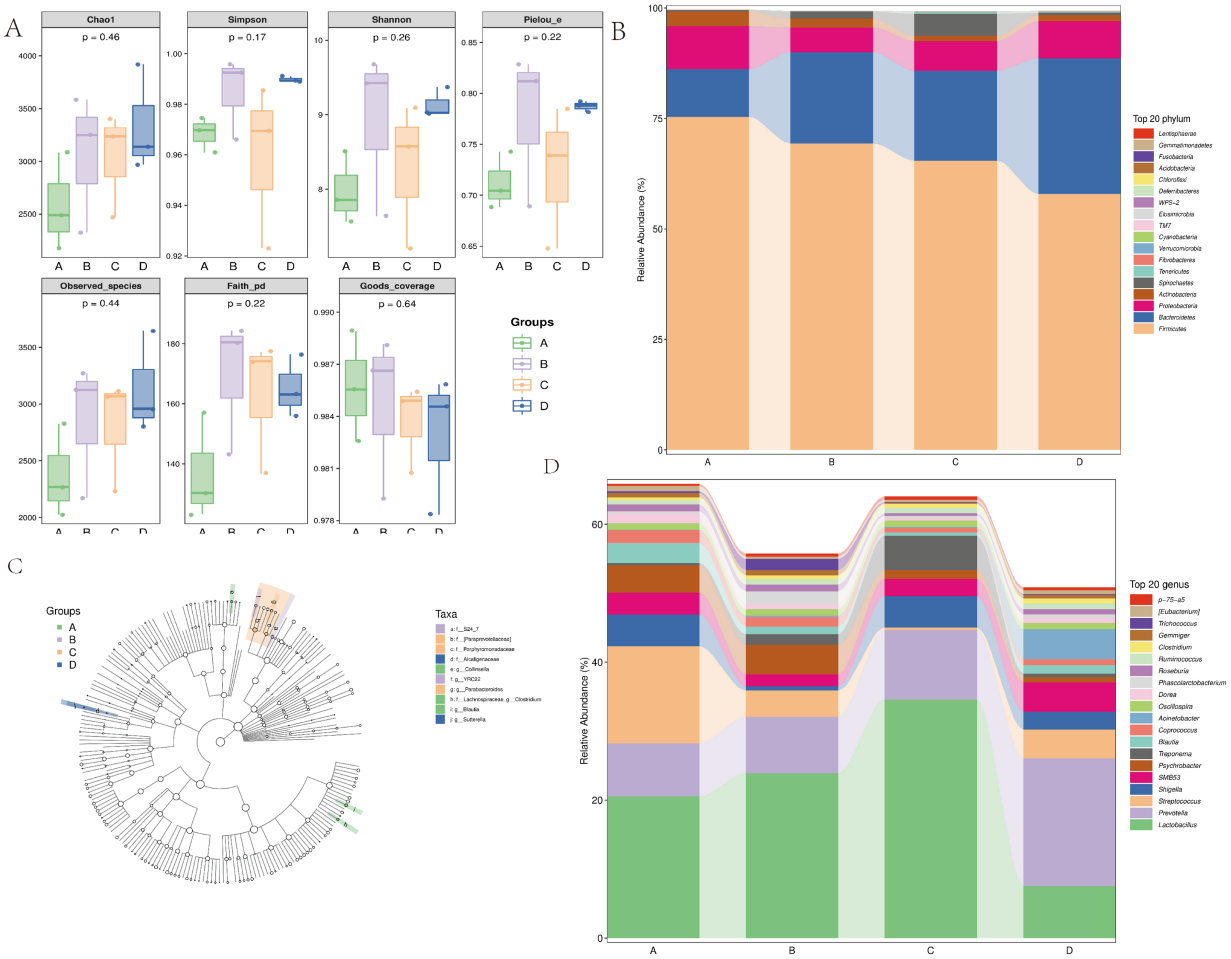
**(A)** Species community richness indices form the 10% alfalfa meal group **(B)**, CON group (A), 20% alfalfa meal group (C), and 30% alfalfa meal group (D). **(B–D)** The taxonomic profiles obtained in the 16S rRNA gene sequencing of sow fecal bacteria, during the gestation period, for the CON group and the 10, 20, and 30% alfalfa meal groups. B presents the relative abundance of the top 10 phyla. C presents the relative abundance of the top 10 genera.

The top 10 phyla and top 10 genera in terms of the relative abundance of fecal microbiota present in Songliao sows are depicted in Figure 7. Longer columns were observed at the genus and species levels while shorter columns were observed at the phylum level, indicating the higher resolution of annotations. As presented in Figures 8B,D, Bacteroidetes and Firmicutes were the most dominant phyla in A, B, C, and D, followed by Proteobacteria, Spirochaetes, and Actinobacteria. At the genus level, *Lactobacillus* was the most dominant genus in all four treatment groups. The other major genera included *Prevotella*, *Ruminococcus*, *Streptococcus*, *Treponema*, *Blautia*, and *Clostridium*. These observations suggested that the abundance of fecal microbial communities in the CON group was significantly different from that in B, C, and D, which possibly affected host metabolism.

Family Alpha-diversity is an important measure to reflect the richness and diversity of fecal bacterial communities. This measure includes the observed species, Chao1, and Shannon indices. In the present study, a higher *α*-diversity and bacterial abundance were noted in the B group (with 10% alfalfa meal) compared to the other groups (Figure 8A).

The microbial compositions of the fecal samples from the sows in the four treatment groups were further analyzed using the linear discriminant analysis coupled with effect size (LEfSe). The S24_7 family and the YRRC_22 genus were significantly enriched in the 10% alfalfa meal group. The Paraprevotellaceae family and the *Parabacteroides* genus were significantly enriched in the 20% alfalfa meal group. The Alcaligenaceae family and the *Sutterella* genus were significantly enriched in the 30% alfalfa meal group. *Blautia*, *Collinsella*, and *Clostridium* were enriched in the no alfalfa meal group at the genus level (Figure 8C). These results indicated that with the increase in the proportion of alfalfa in the diets of sows, the composition of intestinal microbial communities in Songliao sows was altered significantly during the late pregnancy period.

**Figure 9:**
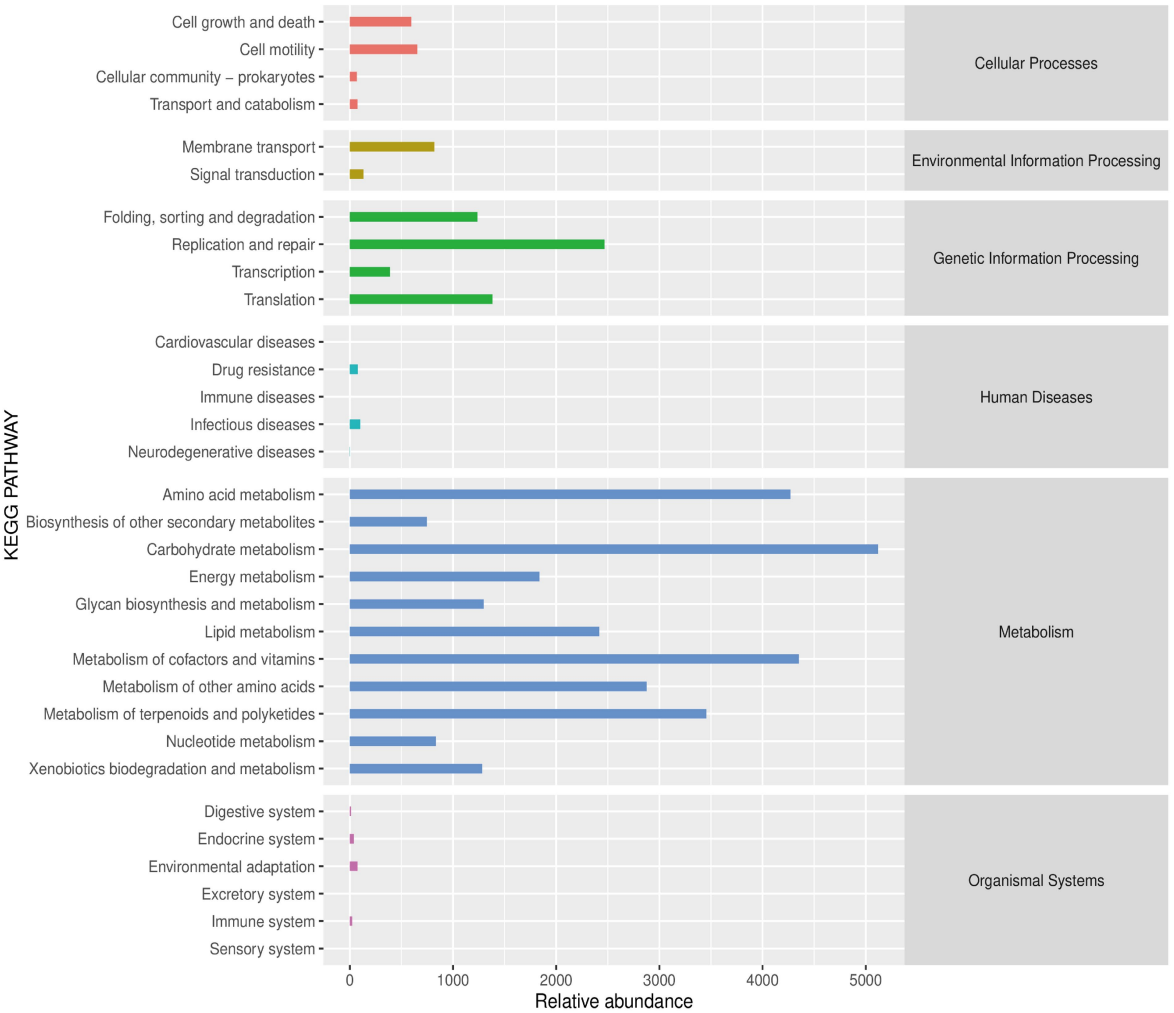
The predicted abundance map of the secondary functional KEGG pathways.

Functional analyses revealed that the microbial communities were mainly enriched in several signaling pathways associated with metabolism, such as carbohydrate metabolism, lipid metabolism, and amino acid metabolism (Figure 9).

### 3.6 Correlation analysis revealed the potential interactions between the microbiota and metabolites

**Figure 10:**
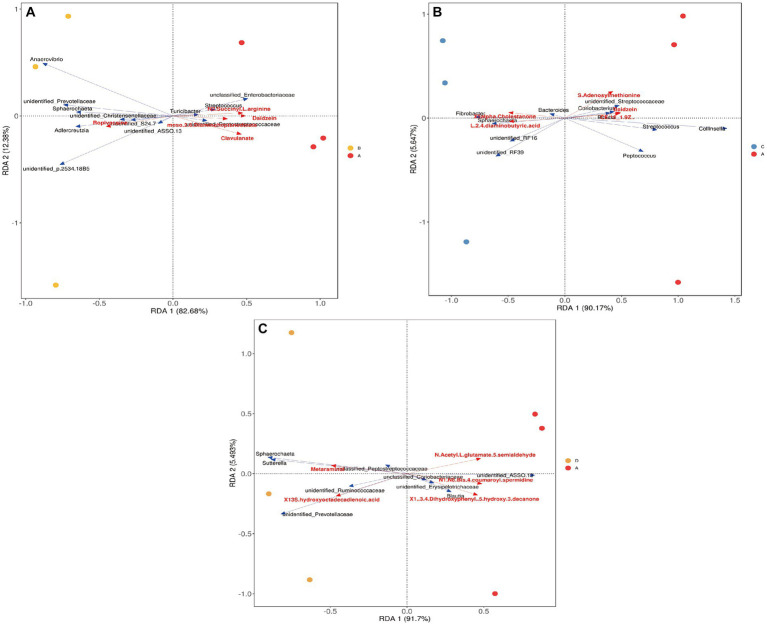
Redundancy analysis results of differential metabolites and microbiota. **(A)** CON group (A)VS 10% alfalfa meal group, **(B)** CON group (A)VS 20% alfalfa meal group, **(C)** CON group (A)VS 30% alfalfa meal group **(D)**. The red arrow indicates metabolites. The blue arrow indicates the microbiota. An acute angle indicates a positive correlation between the metabolites and the microbiota while an obtuse angle indicates a negative correlation.

Recent evidence suggests that the gut microbiota may promote host physiology through the production of various metabolites (Krautkramer et al., 2021), and these metabolites, serving as epigenetic regulators, have a complicated relationship with host genetics and microbiota (Woo and Alenghat, 2022). In the present study, the intestinal microbiota composition and metabolism of Songliao sows during late gestation were revealed to be significantly altered upon feeding the sows with meals containing dietary fiber. A correlation analysis was performed to evaluate the potential relationship between the altered gut microbiota composition and different metabolites. As shown in Figure 10A, the S24_7 family and the *Sphaerochaeta* genus were negatively correlated to Daidzein in the 10% alfalfa meal group compared to the no alfalfa meal group (*p <* 0.001). In addition, the *Sphaerochaeta* genus was negatively correlated to Erucic acid (*p <* 0.001), and the *Prevotellaceae* genus was negatively correlated to Harmaline in the 10% alfalfa meal group compared to the no alfalfa meal group (*p <* 0.001). As visible in Figure 10A, the *Blautia* genus was positively correlated to Daidzein (*p <* 0.001). In Figure 10B, it is evident that the *Fibrobacter* genus was positively correlated to 5-alpha-cholestanone while *Streptococcus* was negatively correlated to 5-alpha-cholestanone in the 20% alfalfa meal group compared to the no alfalfa meal group (*p <* 0.001). As shown in Figure 10C, the *Sphaerochaeta* and *Sutterella* genera were positively correlated to Metaraminol in the 30% alfalfa meal group compared to the no alfalfa meal group (*p <* 0.001).

## 4 Discussion

Dietary fiber is a vital nutrition factor, which plays a key role in maintaining intestinal function in pregnant sows (Hu et al., 2023). In the present study, the weight gain of piglets at birth was observed to be higher in the sows fed with diets containing 10% alfalfa compared to those fed with the CON group diet (*p <* 0.05). Similarly, in a previous study, sows fed a diet with a higher content of dietary fiber during the gestation stage presented a higher litter weight than the sows fed diets with a low dietary fiber content (Che et al., 2011). In the present study, dietary supplementation with 10, 20, and 30% alfalfa significantly increased the concentration of GSH compared to no dietary supplementation with alfalfa. Interestingly, as the proportion of alfalfa increased, the concentration of SOD significantly increased, and the MDA levels decreased. It was speculated that diets with a higher dietary fiber content maintained the oxidative balance system in the body of sows. In a previous study, serum biochemical parameters were evaluated to assess the physiological and metabolic status of the animals (Balan et al., 2019), and it was discovered that sex differences led to different levels of serum total cholesterol and triglyceride in Meishan pigs. Glycerol-3-phosphate acyltransferase 1 (GPAT1) is a rate-limiting enzyme for TG biosynthesis, and levels are closely correlated to sex differences (male < female) in the serum TG levels of Meishan pigs (Kojima and Degawa, 2024). Bamboo shoot fiber supplementation in the diet beginning from day 65 of gestation to farrowing reportedly improved the reproductive performance of sows by improving their insulin sensitivity (Huang et al., 2023). In the present study, sows fed with different levels of dietary fiber exhibited increased serum levels of TC and Glucose. However, in a previous study, the levels of cholesterol, triglyceride, and estradiol in the gilts (age 205 days) fed with soluble fiber (SF) were lower (Zhou et al., 2021). It was speculated that the role of dietary fiber in regulating the reproductive performance of Songliao sows, as observed in the present study, could be related to the breed characteristics and physicochemical of the sows. A previous study demonstrated that dietary SF consistently lowered plasma LDL cholesterol (LDL-C) concentrations (Roy et al., 2002). In the present study, the decreased LDL-C levels in the alfalfa addition group could be related to the increased mobilization of the body reserves of sows.

Furthermore, the sows fed with different levels of dietary fiber exhibited an increased abundance of *Lactobacillus*, *Clostridiales*, and *Firmicutes* in the present study. Evidence suggests that Lactobacillus johnsonii rescue could restore decreased SCFAs (mainly acetate and butyrate) and colonic barrier damage in high-copper-induced intestinal damage of pig model (Wen et al., 2023), indicating that *Lactobacillus*, as a novel type of probiotic, promotes intestinal health during the gestation period. It is well-recognized that *Clostridium*, as a Gram-positive bacillus, colonizes the intestines of animals. *Clostridium butyricum* regulates the chemical barrier, immune barrier, and microbial barrier functions in pigs, thereby playing a vital role in energy metabolism and the development of normal intestinal epithelial cells (Tang, 2024). Probiotics reportedly increased the total antioxidant capacity of muscles after nerve injury in mice, implying that probiotics could accelerate muscle regeneration via the gut–brain axis (Jabeen et al., 2023). Probiotics may be considered neuroprotective agents, providing protection against intestinal microbial disorders (Hou et al., 2022). Accordingly, it was speculated that the sows fed with different levels of dietary fiber in the present study might have increased their intestinal microbial abundance to prevent the occurrence of intestinal disorders. Dietary fiber is considered a probiotic essential for improving gut function, and dietary fiber types with different structural properties exert different effects on the proliferation of bifidobacteria (Wang H. et al., 2022). Non-digestible fiber is decomposed by bacteria, producing a range of energy substrates, which act as bacteria-derived chemical signals that activate enteroendocrine cells to release peptide Y Y (PYY) and glucagon-like peptide-1 (GLP-1) (Fetissov, 2017). Studies have indicated that PPY plays a role in maintaining fungal balance within the mammalian digestive system. PPY serves as an antimicrobial peptide (AMP) that contributes to the maintenance of gut fungal commensalism (Pierre et al., 2023). Accordingly, it was speculated that sows fed with diets containing alfalfa might have improved their reproductive performance by increasing the abundance of intestinal probiotics, which were controlled by the physicochemical properties of the dietary fiber.

It is becoming increasingly evident that gut microbes serve as metabolic substrates to regulate gene expressions (Yang et al., 2022). Microbiota-derived metabolites serving as epigenetic substrates and enzymatic regulators caused changes in the chromatin (Woo and Alenghat, 2022; Wang et al., 2024). Gut microbial metabolites were reported to directly affect the balance of gut microbiota (Guo et al., 2008). In the present study, Firmicutes, Proteobacteria, Spirochaetes, and Actinobacteria were identified as the dominant responders in the gut to the breakdown of dietary fiber at the phylum level, while *Lactobacillus* was the most dominant at the genus level. The other major genera were *Prevotella*, *Ruminococcus*, *Streptococcus*, *Treponema*, *Blautia*, and *Clostridium*. Previous studies have indicated that dietary fiber is broken down by the microbiota and fermented into SCFAs for host utilization. SCFAs may be absorbed directly by the intestinal wall to provide energy to the intestinal epithelial cells (Hu et al., 2023). *Lactobacillus*, considered a potentially beneficial genus of microbes, is positively associated with microbial metabolites related to intestinal barrier function (Zhao et al., 2022). A positive association is also reported between total dietary fiber intake and fecal butyrate (Vinelli et al., 2022). Therefore, it was speculated that under the action of intestinal *Lactobacillus*, dietary fiber is fermented, producing a variety of metabolites, such as SCFAs, including acetate, propionate, and butyrate (Jarrett and Ashworth, 2018; Patil et al., 2020). These metabolites serve as important substrates in gluconeogenesis and participate in the regulation of carbohydrate metabolism (Tian et al., 2020). However, the PPAR signaling pathway was observed to be significantly enriched in the 30% alfalfa meal group, and the results from further analysis indicated that sows fed with higher dietary fiber levels could maintain their body condition during pregnancy and prevented abnormal lipid metabolism (Wang Z. et al., 2022). In summary, the interaction between dietary fiber and microbiota could regulate the metabolism and reproductive performance of sows, and this finding would provide novel insights into the use of dietary fiber in sow diets.

Dietary fiber is reported to have a beneficial effect on a range of health issues; for example, it improves glucose tolerance and reduces the glucose response after a meal (Boers et al., 2017; Kovatcheva-Datchary et al., 2015). SCFAs, as key bacterial metabolites, mediate the efficient utilization of dietary fiber by the host, while also providing sufficient energy for the flora, reducing the pH in the gut, and improving the acidic environment in the gut (Koh et al., 2016; Holman et al., 2022). Studies have indicated that SCFAs increase the serum levels of GPL-1 and PPY and decrease the mRNA expressions of fatty acid synthase (FAS), acetyl-CoA carboxylase (ACC), and sterol regulatory element binding protein 1c (SREBP-1c) (Jiao et al., 2020). Therefore, it was speculated that dietary supplementation with different levels of high fiber content that may be fermented into SCFAs by the intestinal microbiota might control the body weight via suppressing appetite and modulating energy metabolism.

The present study revealed that downregulated metabolites, including Daidzein, Harmaline, and Erucic acids were enriched in the 10% alfalfa meal group compared to the no alfalfa meal group. The upregulated metabolites gein and isolipurgenin were enriched in the10% alfalfa meal group, 5-alpha-cholestanone and 2-hydroxadipic acid were enriched in the 20% alfalfa meal group, and sulfoacetalehyde and metaraminol were enriched in the 30% alfalfa meal group. In addition, 5-alpha-cholestanone was enriched in the steroid biosynthesis signaling pathway. Interestingly, 5-alpha-cholestanone, which belongs to the class of steroids containing the oxhydryl group, might be involved in the synthesis of sex hormones. Accordingly, it was speculated that the sows fed with diets containing 10 and 20% alfalfa exhibited improved reproductive performance through the upregulation of 5-alpha-cholestanone. Studies have demonstrated that the addition of 200 mg/kg of Daidzein to the diet of pigs was beneficial in terms of the antioxidant capacity of pigs (Xiao et al., 2015). Daidzein reportedly protected against H_2_O_2_-induced oxidative stress in IPEC-J2 cells (Li et al., 2022). In the present study, sows fed with different levels of alfalfa exhibited reduced Daidzein content in the feces. In addition, adenosine monophosphate (AMP) was mainly related to the cAMP signaling pathway in sows from the 30% alfalfa meal group. Cyclic adenosine monophosphate (cAMP) was first identified as a secondary messenger and is well-recognized as a key regulator of metabolism and glucose homeostasis in multiple organs (Wang et al., 2021). Therefore, the improvement in the reproductive performance of sows in the 30% alfalfa meal group could be related to the energy metabolism homeostasis of these sows. Studies have indicated that isolipurgenin may relieve exercise-induced fatigue and oxidative stress in mice (Wang and Ma, 2024). Isolipurgenin was also reported to inhibit the PI3K/Akt/mTOR signaling pathway and enhance apoptosis in endometrial cancer cells (Wang and Ye, 2023). In the present study, isolipurgenin was upregulated in the sows fed with high-fiber diets. These observations implied that isolipurgenin might be contributing to improving the reproductive performance of sows by decreasing oxidative stress and preventing the occurrence of reproductive diseases. Erucic acid (EA) is a monounsaturated fatty acid derived from the *Brassicaceae* family. Mounting evidence suggests that erucic acid exhibits cardiotoxic effects in rats (Galanty et al., 2023). EA reportedly exerted a greater negative effect on the laying performance of ducks by affecting the gut microbiota, lipid metabolism, and follicular atresia (Tan, 2022). Therefore, it was speculated that dietary supplementation with high fiber could reduce the production of intestinal toxic metabolites, thereby maintaining a good physiological state of sows during pregnancy.

The correlation analyses conducted in the present study revealed the potential interactions between the microbiota and metabolites. Studies have indicated that *Ruminococcaceae* are responsible for the breakdown of various polysaccharides and fibers (Shang et al., 2016). In addition, the relative abundance of *Ruminococcaceae* exhibited a significant negative correlation to the metabolic disease of the liver (Dinan and Cryan, 2017). In the present study, *Ruminococcaceae* was revealed as the dominant bacterial group at the genus level, which might be a contributor to maintaining normal liver functioning. Spearman correlation analysis revealed that *Fibrobacter* was positively correlated to 5-alpha-Cholestanone. *Fibrobacter succinogenes* is a dominant species in the gastrointestinal tract microbiota in animals, especially in the rumen, which is an essential site for the breakdown of dietary fiber (Kobayashi et al., 2008). Moreover, diets containing higher fiber levels inhibited energy deposition in most cases and improved the reproductive performance of sows (Li et al., 2021). Cholesterol is used as a precursor in the synthesis of reproduction-related hormones. It was, therefore, speculated that dietary fiber is broken down by gut microbes, producing the precursors for the synthesis of hormones related to reproduction. In addition, it was discovered that dietary fiber consumption improves insulin sensitivity, changes the tryptophan metabolism, and increases the abundance of *Sphaerochaeta* (Li Y. et al., 2024). In mammals, insulin sensitivity increases gradually during the third trimester (30–70%) to meet the metabolic requirements of the mother and provide adequate glucose for fetal growth and development (Liu et al., 2020). Metaraminol has a similar pressor effect to norepinephrine in septic shock; it does not increase the heart rate or aggravate kidney injury after shock compared to norepinephrine (Li X. et al., 2024). In the present study, the abundance of *Sphaerochaeta* was negatively correlated to Metaraminol. Therefore, it was speculated that dietary fiber might contribute to increasing the production of SCFAs under the action of gut microbes, thereby increasing the insulin sensitivity of sows and providing energy sources for fetal growth and development. SCFAs produced upon the microbial fermentation of dietary fiber might have an important role in maintaining maternal physiological homeostasis, which implies that microbial metabolites, such as acetate and butyrate, could provide energy for the proliferation of porcine intestinal epithelial cells. A previous study on rats revealed *Prevotellaceae* as the most abundant ASV (amplicon sequence variant) under cold stress and as the mouse gut microbes that regulate fat accumulation by promoting thermogenesis (Ziętak et al., 2016). In the present study, *Prevotellaceae* were revealed to be negatively correlated to Harmaline. Harmaline was reported to strongly inhibit the uptake of phenylalanine in the guinea pig intestine *in vitro* (Sepúlveda and Robinson, 1975). *Parabacillus* is a Gram-negative bacterium that is vital to anti-inflammatory effects and glucose and lipid metabolism (Wang et al., 2019). Therefore, it is understood that a complex network of mutual constraints exists between the intestinal microbes and metabolites in animals. Collectively, the above findings suggest that dietary fiber improves the reproductive performance of sows by regulating the abundance of gut microbiota and metabolite production in sows. However, the mechanism underlying the interaction between sow metabolites and gut microbiota has to be investigated further.

## Data Availability

The data presented in the study are deposited in the NCBI repository (https://www.ncbi.nlm.nih.gov/), accession number PRJNA1184394.
